# Uplink Scheduling and Adjacent-Channel Coupling Loss Analysis for TD-LTE Deployment

**DOI:** 10.1155/2014/685102

**Published:** 2014-02-20

**Authors:** Woon-Young Yeo, Sung Ho Moon, Jae-Hoon Kim

**Affiliations:** ^1^Department of Information and Communication Engineering, Sejong University, 98 Gunja-dong, Gwangjin-gu, Seoul 143-747, Republic of Korea; ^2^Network Technology R&D Center, SK Telecom, 9-1 Sunae-dong, Bundang-gu, Seongnam 463-838, Republic of Korea; ^3^Department of Industrial Engineering, Ajou University, Yeongtong-gu, Suwon 443-749, Republic of Korea

## Abstract

TD-LTE, one of the two duplexing modes in LTE, operates in unpaired spectrum and has the advantages of TDD-based technologies. It is expected that TD-LTE will be more rapidly deployed in near future and most of WiMax operators will upgrade their networks to TD-LTE gradually. Before completely upgrading to TD-LTE, WiMax may coexist with TD-LTE in an adjacent frequency band. In addition, multiple TD-LTE operators may deploy their networks in adjacent bands. When more than one TDD network operates in adjacent frequency bands, severe interference may happen due to adjacent channel interference (ACI) and unsynchronized operations. In this paper, coexistence issues between TD-LTE and other systems are analyzed and coexistence requirements are provided. This paper has three research objectives. First, frame synchronization between TD-LTE and WiMax is discussed by investigating possible combinations of TD-LTE and WiMax configurations. Second, an uplink scheduling algorithm is proposed to utilize a leakage pattern of ACI in synchronized operations. Third, minimum requirements for coexistence in unsynchronized operations are analyzed by introducing a concept of adjacent-channel coupling loss. From the analysis and simulation results, we can see that coexistence of TD-LTE with other TDD systems is feasible if the two networks are synchronized. For the unsynchronized case, some special cell-site engineering techniques may be required to reduce the ACI.

## 1. Introduction

Long Term Evolution (LTE) is a radio platform that allows mobile operators to achieve much higher peak data rates and better spectral efficiency than those of the third generation (3G) networks (e.g., WCDMA and cdma2000) [1]. LTE was initiated by the Third Generation Partnership Project (3GPP) in 2004 and is now commercially deployed or in progress worldwide. The LTE standard has two duplexing modes (LTE-TDD and LTE-FDD) and the technical specification of the two modes is almost the same. The LTE-TDD, also known as TD-LTE, can operate in unpaired spectrum and supports asymmetric resource allocation between uplink and downlink. Even though both TD-LTE and LTE-FDD will be widely used, TD-LTE will be more rapidly deployed in near future due to a number of advantages of the TDD-based technology [2].

As a fourth generation (4G) technology competing with TD-LTE, WiMax provides a broadband wireless access to mobile subscribers based on IEEE 802.16 standards [3]. Starting from IEEE 802.16, the standard was amended many times and changed to 802.16a, 802.16c, 802.16d, 802.16e, and 802.16m. IEEE 802.16e is the most popular standard for WiMax today and 802.16m is the most advanced version approved in March 2010. WiMax networks have been commercially deployed in many countries, but most of these are niche deployments for specialized applications [4]. WiMax operators can upgrade 802.16e to 802.16m or migrate to TD-LTE directly in their unpaired spectrum. Considering the competitive 4G markets, it may be better to upgrade their network towards TD-LTE gradually instead of upgrading to 802.16m.

Upgrading to TD-LTE is a potentially difficult decision for WiMax operators because most WiMax operators have recently launched their networks. Before completely upgrading to TD-LTE, WiMax may coexist with TD-LTE in the same frequency band because the two systems are technically very alike and the mobile operators want to minimize the extra cost for TD-LTE deployment. WiMax Forum has considered a network evolution path to accommodate harmonization and coexistence across multiple wireless access technologies including TD-LTE [5]. In addition, it is also possible that multiple TD-LTE operators deploy their networks in adjacent frequency bands. When two TDD networks operate in adjacent frequency bands and are deployed in the same area, severe interference may happen due to adjacent channel interference and unsynchronized transmission timing between the two networks.

A number of papers and technical reports have been published for coexistence analysis among 3G/4G wireless networks. In [5], network and air interface requirements for WiMax were specified to facilitate the coexistence of WiMax and TD-LTE. In [6], capacity loss of 3G/4G TDD systems (TD-SCDMA, TD-HSDPA, and TD-LTE) was presented when they were interfered by an LTE-Advanced system operating on an adjacent frequency band. In [7], coexistence of TD-LTE and LTE-FDD was analyzed, and intercell interference coordination was studied through simulation. In [8], requirements for coexistence between TD-SCDMA and TD-LTE were given by a simple interference calculation, and the required antenna installation was investigated to reduce mutual interference.

In this paper, coexistence between TD-LTE and other systems is analyzed and operational requirements are provided to maintain an acceptable level of adjacent channel interference. Most of examples and applications will be explained by assuming TD-LTE/WiMax coexistence, but the proposed methodology and analysis can be applied to other coexistence scenarios (e.g., TD-LTE vs. TD-LTE and TD-LTE vs. LTE-FDD). There are three research objectives in this paper. First, frame synchronization between TD-LTE and WiMax is discussed by investigating possible combinations of TD-LTE and WiMax configurations. Because an unsynchronized operation may cause serious interference, the synchronization feasibility between the two systems is checked and some additional techniques for synchronization are explained. Second, an uplink scheduling algorithm is proposed to make the uplink transmission robust against the adjacent channel interference when two networks are synchronized. Third, a concept of adjacent-channel coupling loss is introduced to estimate the minimum requirements for network coexistence when two networks are not synchronized. Because there has been little work on the interference analysis for the unsynchronized operation, the proposed analysis methodology can provide a guideline for cell-site engineering in the unsynchronized network.

The rest of this paper is organized as follows. The basic frame structures of TD-LTE and WiMax are explained in Section 2. In Section 3, the synchronization feasibility between TD-LTE and WiMax is analyzed by checking possible combinations of network configurations and by adjusting some system parameters. In Section 4, an uplink scheduling algorithm is proposed for robust operations in the synchronized operation, and a concept of adjacent-channel coupling loss is introduced to estimate the coexistence requirements in the unsynchronized operation. In Section 5, evaluation methodology and simulation assumptions are described in detail. The simulation and analysis results are presented and discussed in Section 6. Finally, Section 7 summarizes and concludes this paper.

## 2. TD-LTE and WiMax Frame Structure

Downlink (DL) and uplink (UL) transmissions in TD-LTE and WiMax are based on orthogonal frequency division multiple access (OFDMA). Specifically, OFDMA is used for DL of TD-LTE and for UL and DL of WiMax. The UL transmission for TD-LTE uses a technology called single carrier-frequency division multiple access (SC-FDMA), which is similar to OFDMA but more power efficient than OFDMA at a user equipment (UE). In this section, frame structures of the two systems are explained because the difference between them is critical to analyze the network synchronization.

### 2.1. TD-LTE Frame Structure

In the 3GPP specification, there is no operational difference between TD-LTE and LTE-FDD at higher layers or in the system architecture [2]. The design goal of the LTE physical layer is to achieve as much commonality as possible. As in LTE-FDD, a resource block (RB, 180 kHz) is a minimum allocation unit in frequency domain and defined as a group of 12 consecutive subcarriers with subcarrier spacing of 15 kHz. The minimum allocation unit in time domain is a subframe or transmission time interval (TTI), which has a duration of 1 ms. One subframe consists of two time slots and has 12 or 14 OFDM symbols depending on a length of cyclic prefix (CP).

**Figure 1:**
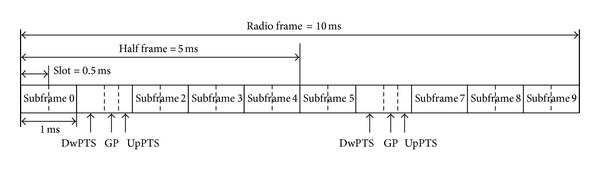
TD-LTE frame structure (5 ms periodicity).

The TD-LTE frame structure is shown in Figure 1. TD-LTE has a 10 ms TDD frame structure. A radio frame consists of 10 subframes. Subframes 0 and 5 contain synchronization signal and broadcast information necessary for a UE to perform synchronization and obtain relevant system information. Subframes 1 and 6 are special subframes that serve as a switching point between DL and UL transmission. The special subframe contains three fields: downlink pilot time slot (DwPTS), guard period (GP), and uplink pilot time slot (UpPTS).

Table 1 provides the special subframe configurations, where the length of each field is given in multiples of OFDM symbols and a normal CP is assumed. DwPTS can be viewed as an ordinary DL subframe and is used for DL data transmission. Unlike a normal subframe, where the control region can span up to three OFDM symbols, the maximum control region in DwPTS is limited to two OFDM symbols. UpPTS has a short duration of one or two OFDM symbols and can be used for transmission of sounding reference signals (SRS) and random access. SRS enables a base station (BS) to estimate UL channel quality. SRS can be also provided by normal subframes as in LTE-FDD. Random access typically uses one of normal subframes, enabling a relatively long random access preamble. GP is used to prevent overlap between transmission and reception, and the length of GP depends on the cell size. Because formats 0 and 5 have a large GP, they can support a large cell size, but they cannot provide additional DL capacity due to a small DwPTS.

In TD-LTE, two switching periodicities are supported: 5 ms and 10 ms. For the 5 ms periodicity, subframe 6 is a special subframe identical to subframe 1, whereas it is a regular DL subframe for the 10 ms periodicity.  Table 2 shows the DL/UL allocation according to the switching periodicity. In this paper, TD-LTE configurations 0 to 2 are candidates for network coexistence because WiMax only supports a frame length of 5 ms.

### 2.2. Frame Structure of WiMax IEEE 802.16e-TDD

**Figure 2:**
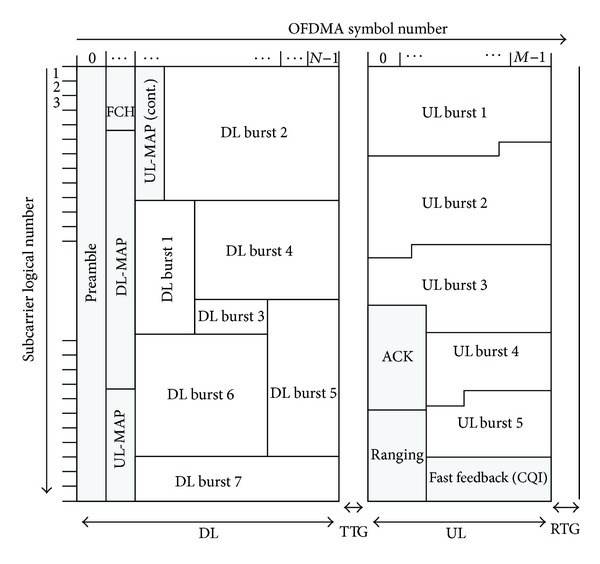
Frame structure of IEEE 802.16e-TDD.

Figure 2 illustrates a mobile WiMax (IEEE 802.16e-TDD) frame structure [3]. Each 5 ms frame is time-division duplexed with DL and UL subframes. There are time gaps between DL and UL subframes, considering mobile transceiver turnaround time and a guard time avoiding interference between DL and UL signals. A time gap for transition from DL to UL subframe is called transmit time gap (TTG). A time gap for transition from UL to DL subframe is called receive time gap (RTG).

At the beginning of each frame, DL control information is transmitted and consists of a preamble, a frame control header (FCH), and MAP messages. The preamble can be used for synchronization and DL channel estimation. The subcarriers allocated to the preamble are uniformly distributed over the spectrum and occupy every third subcarrier. FCH provides information required to decode the subsequent DL-MAP message. The DL/UL-MAP messages indicate the resource allocation for DL/UL data and control transmission. UL control channels consist of ranging, channel quality indicator (CQI), and acknowledgment (ACK) channels. The ranging channel provides the random access for initial entry, timing adjustment, periodic synchronization, bandwidth request, and handover entry. The CQI or fast-feedback channel is used by a mobile station to report the measured signal quality back to an access point. The ACK channel reports ACK/NACK feedback for DL data transmission.

The WiMax system supports a scalable system bandwidth of 3.5, 5, 7, 8.75, and 10 MHz. If TTG and RTG are excluded, each 5 ms frame has 47 OFDM symbols for 5 and 10 MHz bandwidth, 42 symbols for 8.75 MHz bandwidth, and 33 symbols for 3.5 and 7 MHz bandwidth [9]. In addition, there are different numbers of DL/UL ratios depending on the system bandwidth: 10 different DL/UL ratios for 5 and 10 MHz bandwidth and 7 ratios for 3.5, 7, and 3.75 MHz bandwidth. The DL/UL ratio is adjustable to support asymmetric traffic, and the parameters for 10 MHz bandwidth are summarized in Table 3. Internationally, the 29 : 18 DL/UL ratio is very common and popular for WiMax operators. Note that the ratio of DL and UL may not dynamically change per cell but should change on a system-wide basis.

## 3. Frame Synchronization Analysis for TD-LTE and WiMax Coexistence

All cellular wireless systems suffer from interference in adjacent bands and TDD can have two more serious interference sources: from BS to BS and from UE to UE. Depending on frequency arrangements, the interference may occur between two or more TDD systems or between TDD and FDD systems. TDD performance is significantly degraded when DL and UL signals from two TDD systems operating in adjacent bands overlap in time. Special cell-site engineering techniques may be required to reduce the excessive interference. The simplest way is frame synchronization, where DL/UL frame boundaries across TDD networks are aligned in time. Frame start timings of all BSs can be synchronized through GPS, IEEE 1588 version 2, and “network listening” [9].

**Figure 3:**
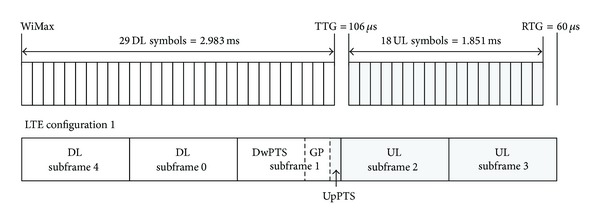
Example of frame synchronization between TD-LTE and WiMax.

Considering frame structures of TD-LTE and WiMax, it is necessary to specify a frame offset to TD-LTE. The frame offset ensures that the start of continuous DL subframes lines up with the WiMax DL subframe.  Figure 3 shows an example of the frame synchronization between TD-LTE and WiMax, assuming TD-LTE with configuration 1 and WiMax with a default frame configuration of (29, 18). In this example, UpPTS of TD-LTE overlaps with the last DL symbol of WiMax, and thus one DL symbol of WiMax and UpPTS of TD-LTE may cause serious interference to each other if they operate in adjacent bands.

In this section, frame synchronization between TD-LTE and WiMax is analyzed for possible combinations of system configurations. In TD-LTE, configurations 0 to 2 in Table 2 are candidates for coexistence due to a frame length of WiMax. WiMax has up to 10 different DL/UL ratios and the number of OFDM symbols per frame is different depending on the system bandwidth. The 10 MHz WiMax system is assumed in this analysis, but it is possible to apply the same analysis technique to other bandwidths.

In TD-LTE configuration 0 (DL : UL = 1 : 3), one subframe is configured for DL transmission in a 5 ms frame. Because the WiMax DL has more OFDM symbols than UL, it is difficult to align the WiMax frame to TD-LTE. The last part of WiMax DL always overlaps with the UL subframe of TD-LTE. In all combinations, frame synchronization is not supported between the two systems, and thus TD-LTE configuration 0 is not suitable for coexistence in a normal network environment.

As for TD-LTE configuration 1 (DL : UL = 2 : 2), frame synchronization between the two TDD systems is possible in some combinations, but the default WiMax configuration of (29, 18) overlaps with UpPTS as shown in Figure 3. To solve the synchronization problem for the default configuration, it has been suggested to disable some resources in one of the two systems, by disabling the last one or two symbols in WiMax or by disabling UpPTS [9]. Due to capacity loss of WiMax, it is desirable to disable UpPTS instead of WiMax DL symbols. Note that UpPTS is used to transmit RACH and SRS, and UL symbols in a normal subframe can be allocated to RACH and SRS. Tables 4(a) and 4(b) show the number of overlapped TD-LTE symbols when UpPTS is enabled and disabled, respectively. The overlap region occurs when the last DL symbols of WiMax overlap with subframe 2 (UL) of TD-LTE or the first UL symbols of WiMax overlap with DwPTS. If UpPTS is enabled, three WiMax configurations can support frame synchronization with TD-LTE, whereas four WiMax configurations can do by means of disabling UpPTS.

In TD-LTE configuration 2 (DL : UL = 3 : 1), frame synchronization is possible in some combinations, but there is no TD-LTE frame configuration that naturally aligns with the default WiMax configuration of (29, 18). The overlap region occurs mainly because WiMax UL overlaps with DwPTS. If DwPTS is disabled, more WiMax configurations can be synchronized with TD-LTE. Tables 5(a) and 5(b) show the number of overlapped TD-LTE symbols when DwPTS is enabled and disabled, respectively. With enabled DwPTS, frame synchronization is only supported in a few combinations. TD-LTE special subframe formats 0 and 5 have a large GP supporting a large cell size and WiMax configuration of (35, 12) allocates much more DL resources than UL. These network parameters are not commonly used in commercial TD-LTE and WiMax services. If DwPTS is disabled, the first seven WiMax configurations can support frame synchronization and the last three WiMax combinations partially overlap with TD-LTE subframe 1 (DL). However, because DwPTS can be used for DL data transmission in TD-LTE, disabling DwPTS can result in up to 22% performance loss assuming two control symbols in a subframe. Thus, TD-LTE configuration 2 is not strongly recommended for frame synchronization between TD-LTE and WiMax.

## 4. Uplink Scheduling and Adjacent-Channel Coupling Loss Analysis

To analyze coexistence issues, this paper focuses on adjacent channel interference from an aggressor system (WiMax or TD-LTE) to a victim system (TD-LTE) in macrocellular scenarios. The adjacent channel interference (ACI) is the total interference from adjacent channels and mainly related to the amount of signal leakage from a transmitter, the amount of signal loss between two transceivers (i.e., coupling loss), and the ability of a receiver to suppress out of band interference [10]. The adjacent channel leakage is measured in terms of adjacent channel leakage ratio (ACLR), which is defined as the ratio of the desired signal power in its channel to the power measured in an adjacent channel. Similarly, adjacent channel selectivity (ACS) is a measure of the ability of a receiver to filter and reject the signal from adjacent channels. ACS is defined as the ratio of the receiver filter attenuation on the desired channel to the receiver filter attenuation on the adjacent channel(s). The coupling loss (CL) is simply the amount of signal attenuation between the transmitter and receiver. The CL is the sum of the path attenuation (or path loss), antenna gains, and any other cabling losses.

A parameter named ACIR (adjacent channel interference ratio) is used to measure the overall ACI and defined as the ratio of the total power transmitted from an aggressor transmitter to the total interference power affecting a victim receiver in the adjacent channel. The ACIR is expressed as a combination of ACLR and ACS according to the following equation (expressed in linear scale):

(1) ACIR=1(1/ACLR)+(1/ACS).

In (1), the UL limiting factor is the UE transmitter because UE ACLR is much lower than BS ACS (i.e., UE ACLR ≪ BS ACS). Similarly, the DL limiting factor is the UE receiver because UE ACS is much lower than BS ACLR (i.e., UE ACS ≪ BS ACLR) [11, 12].

In this section, an uplink scheduling algorithm is proposed to make the UL transmission robust against the ACI when two different networks are synchronized. In addition, a concept of adjacent-channel coupling loss is introduced to analyze the minimum requirements for network coexistence when two networks are not synchronized.

### 4.1. Uplink Scheduling for Synchronized Operations

**Figure 4:**
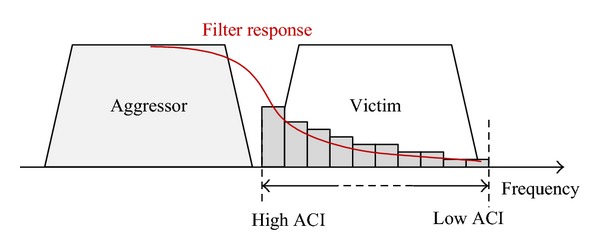
Conceptual example of adjacent channel interference caused by ACLR.

Considering UL ACIR in (1), the limiting factor for UL is UE ACLR, and thus the UL performance loss may depend on the ACLR pattern of aggressor UEs. Generally, the amount of ACI to a victim channel is related to the frequency location of the victim channel. This is also reflected in the UL interference model of 3GPP [12]. The typical UL ACI characteristics are shown in Figure 4 assuming that an aggressor frequency band is just below the victim band. If one of victim channels is located near the upper edge of the aggressor band, it can suffer from more ACI than other channels far from the aggressor band.

Because the ACI pattern is not constant over the victim spectrum, the performance loss can be related to UL frequency allocation policy. For example, if low-ACI RBs are allocated to UEs with low signal quality (i.e., edge UEs), the edge throughput degradation is not so significant even though the cell throughput can be reduced. Similarly, if the low-ACI RBs are allocated to UEs with high signal quality (i.e., central UEs), the cell throughput degradation is negligible by the sacrifice of the edge throughput.

In order to design a robust TDD system against the ACI from the aggressor, it is worthwhile to analyze the UL scheduling algorithms that take into account the ACI distribution over the victim spectrum. The proposed UL scheduling is based on the ACI distribution and is called ACI-based uplink scheduling in this paper. Depending on the allocation policy of the low-ACI RBs, the proposed scheduling has two types of operation: minimum CL first (minCL) and maximum CL first (maxCL). The ACI-based minCL scheduling allocates the low-ACI RBs to the UEs with a low CL (i.e., central UEs), whereas ACI-based maxCL scheduling allocates them to the UEs with a high CL (i.e., edge UEs).

The ACI-based minCL scheduling is expected to minimize the cell throughput loss caused by the ACI. If *N* UEs are selected for UL scheduling, the minCL procedures are described as follows.

 Step  0: divide UL frequency resources into *N* consecutive RB sets to support SC-FDMA transmission. Let *L*(*k*) be a CL between the BS and UE *k*  (*k* ∈ *U* = {1,…, *N*}), and let *I*(*k*) be an ACI level for RB set *k*  (*k* ∈ *R* = {1, …, *N*}) at the BS receiver.
 Step  1: find a UE with the minimum CL to the serving BS in *U*. Letting *i* be the corresponding UE identity,
  (2) $$\begin{array}{c} i=\arg\;\underset{k\in U}{\min\;}L\left(k\right). \end{array}$$
 Step  2: find an RB set with the minimum ACI in *R*. Letting *j* be the corresponding RB set identity,
  (3) $$\begin{array}{c} j=\arg\underset{k\in R}{\min\;}I\left(k\right). \end{array}$$
 Step  3: allocate RB set *j* to UE *i*.
 Step  4: delete *i* in *U* and delete *j* in *R*.
 Repeat Steps 1 to 4 until all RB sets are allocated.

Note that the ACI distribution over the victim spectrum may be different according to frequency planning of mobile operators. If a victim band is located in the middle of two aggressor bands, the ACI pattern may be different from Figure 4.

Another scheduling option, ACI-based maxCL scheduling, is expected to minimize the edge throughput loss because it allocates low-ACI RBs to edge UEs. Procedures for ACI-based maxCL scheduling are almost the same as those of minCL scheduling, except in Step  1. Step  1 and (2) are replaced by the following procedure.

 Step  1: find a UE with the maximum CL to the serving BS in *U*. Letting *i* be the corresponding UE identity,
  (4) $$\begin{array}{c} i=\arg\;\underset{k\in U}{\max\;}L\left(k\right). \end{array}$$

### 4.2. Adjacent-Channel Coupling Loss Analysis for Unsynchronized Operations

It is generally assumed that two TDD systems in adjacent bands are perfectly synchronized, but it is necessary to analyze the unsynchronized network for considering the worst-case scenarios. The interference between a BS and a UE operating in adjacent bands is not significant because the CL between the BS and the UE is high [10]. In the unsynchronized network, TDD can give rise to two more interference mechanisms, between two BSs (BS-to-BS) or two UEs (UE-to-UE). In this section, a concept of adjacent-channel coupling loss is introduced to estimate the minimum requirements for coexistence in the unsynchronized network.

As mentioned before, the CL is the amount of signal attenuation between the transmitter and receiver and is the sum of the path loss (PL), antenna gains, and any other cabling losses. The minimum coupling loss (MCL) represents the lowest reasonable CL between any two transceivers. In this paper, the concept of CL is extended to effectively evaluate the performance of the unsynchronized network. The adjacent-channel coupling loss (ACCL) is defined as follows:

(5) $$\begin{array}{c} \text{ACCL}=L+\text{ACIR}-G_{a}-G_{v}, \end{array}$$

where *L* is the PL between two BSs or two UEs and *G*_*a*_ and *G*_*v*_ are antenna gains of an aggressor transmitter and a victim receiver, respectively. (Cabling loss and other minor losses are ignored.) The transmitted signal from an aggressor is attenuated by the ACCL and acts as interference to a victim receiver.

In the unsynchronized operation, the nearest aggressor from a victim may cause the most serious interference to the victim receiver if it transmits signals in the opposite direction of the victim. Thus, the nearest interferer may dominate the interference on the victim receiver. In unsynchronized UL, the nearest aggressor BS may be a main source of BS-to-BS interference. Similarly, in unsynchronized DL, the nearest aggressor UE can be considered a main source of UE-to-UE interference.

In the proposed ACCL analysis, the system performance is evaluated by assuming a single dominant aggressor around a victim receiver. Because the ACCL in (5) encompasses all gains and losses, the performance evaluation does not need any detailed information about the aggressor, such as PL, filter characteristics, and antenna patterns. In the ACCL analysis, the performance evaluation procedures are as follows.

 Step  1: assume a single aggressor around a victim receiver.
 Step  2: evaluate the system performance for a specific ACCL.
 Step  3: obtain the minimum ACCL that guarantees an acceptable system performance.
 Step  4: design and optimize the system parameters by using the minimum ACCL.

In Step  4, the minimum ACCL can be used for configuring optimal system parameters. The ACCL in (5) consists of adjustable components. First, PL is related to the distance between two BSs or two UEs. Second, ACIR represents filter characteristics of the transmitter and receiver. Finally, *G*_*a*_ and *G*_*v*_ are related to beam patterns and antenna directions at the transmitter and receiver. To meet the minimum ACCL requirement, the following actions are possible: (1) increase a distance between the aggressor and the victim, (2) improve ACLR and ACS performance of the transmitter and receiver, and (3) adjust antenna direction to ensure the minimum interference.

If the ACIR and antenna gains are known, the minimum PL between the aggressor and victim can be calculated by (5). Then it is possible to approximately estimate the minimum distance by a proper PL formula. There are a lot of PL models in the literature [13] and they should be properly applied to the estimation according to environmental and operational factors. In Section 6, the entire ACCL procedures can be found with typical examples, and two PL models are used for BS-to-BS and UE-to-UE cases, respectively.

## 5. Evaluation Methodology and Simulation Assumptions

The simulation framework is based on the Vienna LTE system level simulator [14] and includes additional procedures and algorithms required for performance evaluation. Most of simulation parameters and assumptions are based on [12]. In this section, evaluation methodology and simulation assumptions are explained.

### 5.1. Network Layout

BSs are placed on a hexagonal grid with intersite distance of 750 m. Each BS has three sectors with directional antennas and there are 19 BS sites (57 sectors) in a single network. For a coordinated network, identical cell layouts are applied and aggressor/victim BSs are colocated at the same sites. For an uncoordinated network, identical cell layouts are applied, but with the worst-case shift between sites. Thus, every site in the aggressor network is located at the cell edge of the victim network. The distance between aggressor and victim BSs is 433 m in the uncoordinated layout.

**Figure 5:**
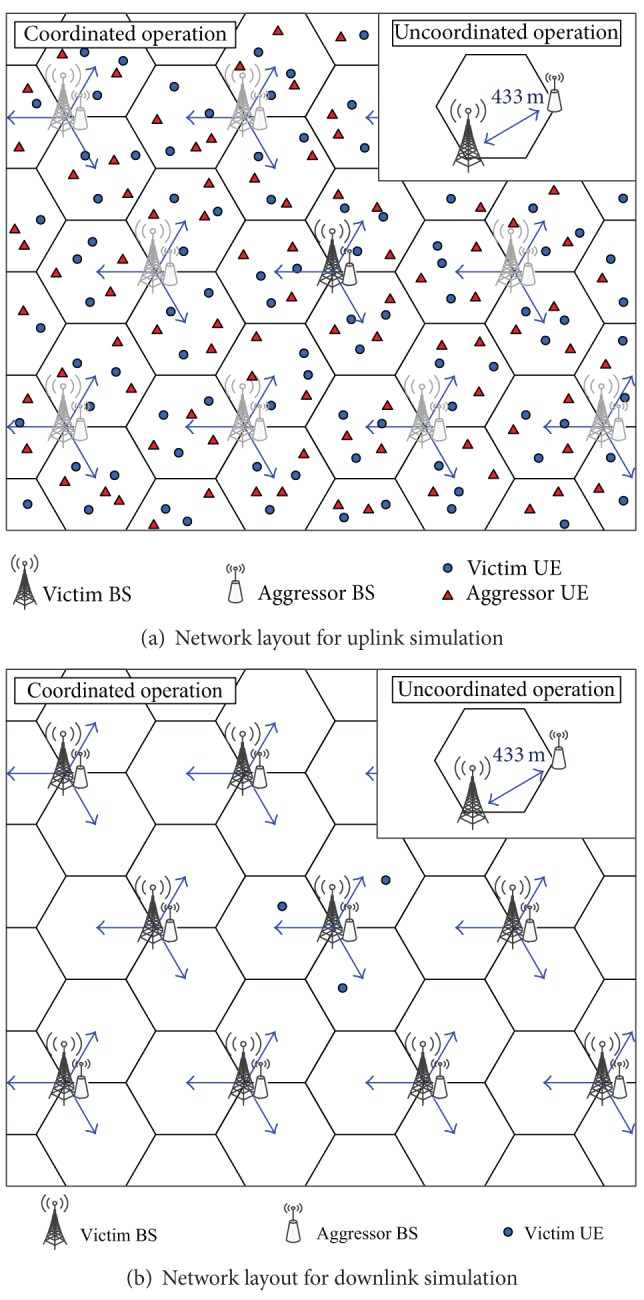
Cell layout for performance evaluation.

In UL simulation, the number of UEs per subframe might affect simulation results because UE transmit power depends on the number of UEs per subframe. In this simulation, three UEs per subframe are assumed and they are randomly distributed in each sector.  Figure 5(a) shows the network layout for the UL simulation, with an example of UE distribution. Circles represent victim UEs in TD-LTE and triangles are aggressor UEs in WiMax or TD-LTE. 1,000 different UE distribution scenarios are applied to the simulation. All BSs of the two TDD systems can control the UE transmit power by a power control mechanism. Statistics are only collected from the central site.

As for DL simulation, the number of UEs per subframe does not affect simulation results because BS transmit power is constant. Only one UE is randomly distributed in each sector.  Figure 5(b) shows the network layout for the DL simulation, with an example of UE distribution. Statistics are only collected from the central site, and three UEs are located in the central site. 1,000 different UE distribution scenarios are applied and all BSs of the two systems transmit at full power of 46 dBm.

### 5.2. Interference Model

In this simulation, the system bandwidth of the victim system (TD-LTE) is 10 MHz and there are 50 RBs in frequency domain. The aggressor system (TD-LTE or WiMax) has the same system bandwidth as the victim system. The frequency band of the aggressor system is adjacent to the victim and its location is just below the victim system in frequency. This interference model is based on [12].

**Figure 6:**
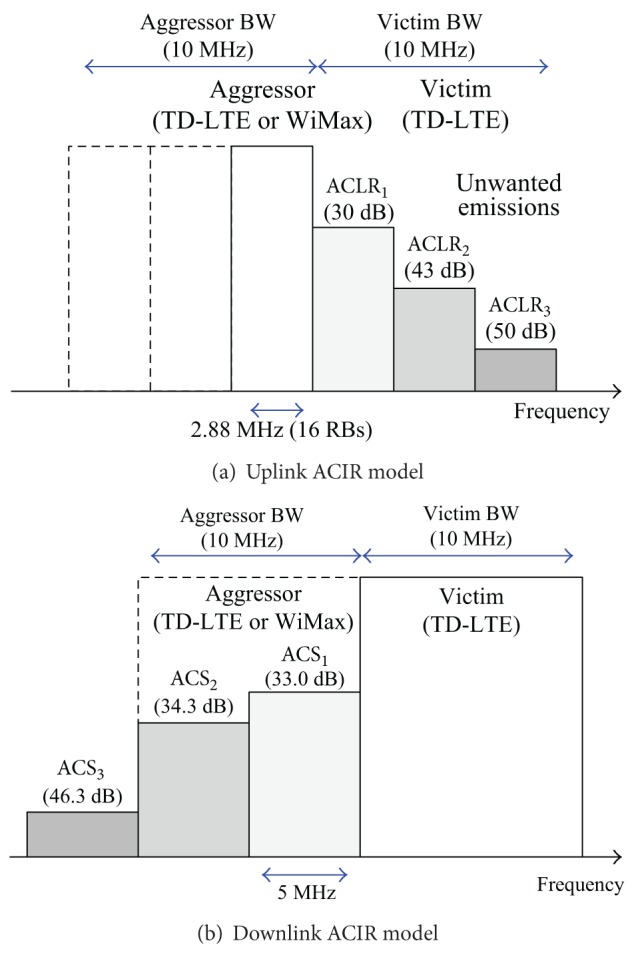
ACIR models for simulation.

For UL, the ACIR is dominated by UE ACLR and UL ACIR *≈* UE ACLR from (1). Three UEs are served simultaneously by round-robin scheduling. Among the 50 RBs, 16 RBs are allocated to each UE and the outer 2 RBs are not used for data transmission. According to [12], the UL ACLR model for 10 MHz bandwidth consists of three emission levels. The UL ACLR model and reference ACLR levels (ACLR_1_, ACLR_2_, and ACLR_3_) are shown in Figure 6(a). If the aggressor UE is adjacent to the victim channel, the ACIR is ACLR_1_ + X dB to the corresponding victim channel, where *X* adjusts the actual ACLR level in simulation. If the aggressor UE is 16 RBs away from the victim channel, the ACIR is ACLR_2_ + X dB. Otherwise, the ACIR is ACLR_3_ + X dB. There are three aggressor UEs in the aggressor band and the resulting ACI is accumulated in each victim UL channel.

A common ACIR for all RBs is used for DL performance evaluation. The ACLR of the aggressor BS has negligible impact on ACIR in (1) and DL ACIR *≈* UE ACS. The DL ACIR model and reference ACS levels (ACS_1_, ACS_2_, and ACS_3_) are shown in Figure 6(b). The common ACIR is mathematically described as ACIR = ACS_avg_ + *X* dB, where ACS_avg_ is the average ACS and *X* is an offset relative to ACS_avg_· ACS_avg_ is calculated by the reference ACS values (expressed in dB scale):

(6) $$\begin{array}{c} 10^{-{\text{ACS}}_{\text{avg}}/10}=\frac{10^{-{\text{ACS}}_{1}/10}+10^{-{\text{ACS}}_{2}/10}}{2}, \end{array}$$

where ACS_3_ is not used in (6) because the aggressor bandwidth is 10 MHz and ACS_3_ is out of range.

### 5.3. Uplink Power Control

In DL, no power control mechanism is applied and transmit power per RB is constant. In UL, the fractional transmit power control (TPC) scheme is adopted [12, 15]. This mechanism will attempt to control the UE transmit power to compensate the PL to the BS. The UE transmit power for a data channel in subframe *i* is set to as follows:

(7) $$\begin{array}{c} P\left(i\right)=\min\left(P_{\max},10\;\text{lo}{\text{g}}_{10}\left(M\left(i\right)\right)+P_{0}+\alpha L\right), \end{array}$$

where *P*_max⁡_ is the maximum transmit power (23 dBm), *L* is the PL between the UE and its serving BS (including antenna gains), and *M*(*i*) is the number of allocated RBs in subframe *i*. In this simulation, there are two TPC sets, 1 and 2, to adjust the UL interference level, and the related parameters are given in Table 6 [12]. Generally, TPC set 1 causes more interference than TPC set 2.

### 5.4. Simulation Parameters and Other Assumptions

The BS antenna radiation pattern to be used for each sector is given as follows:

(8) $$\begin{array}{c} A\left(\theta \right)=-\min\left[12{\left(\frac{\theta }{\theta_{3\;\text{dB}}}\right)}^{2},A_{m}\right],\;\text{where}-180\leq \theta \leq 180, \end{array}$$

where *θ*_3 dB_ is the 3 dB beam width that corresponds to 65 degrees and *A*_*m*_ = 30 dB is the maximum attenuation.

The PL (*L*) is given as follows according to the ITU-R model with high BS antennas [13]:

(9) L=40(1−4×10−3Δhb)log10(d)−18 log10(Δhb)+21 log10(fc)+80,

where *d* is the BS-UE separation in kilometers, *f*_*c*_ is the carrier frequency in MHz, and Δ*h*_*b*_ is the BS antenna height in meters measured from the average rooftop level. Considering a carrier frequency of 2,000 MHz and a BS height of 15 m above average rooftop level, the propagation model is given as follows [12]:

(10) $$\begin{array}{c} L=128.1+37.6\;\text{lo}{\text{g}}_{10}\left(d\right). \end{array}$$

Other simulation parameters are summarized in Table 7. DwPTS can be used for DL data transmission and special subframe format 2 is used. Two OFDM symbols are assigned to DL control signaling in each subframe (including DwPTS). The link-level performance for UL is based on [16]. The retransmissions of UL and DL packets are not considered.

## 6. Results and Discussion

In this section, the performance of the proposed UL scheduling is compared with random scheduling in synchronized operations, and the minimum ACCL is obtained to estimate an acceptable level of interference in unsynchronized operations. The simulation results are presented in terms of throughput reduction relative to the reference throughput without ACI. The cell throughput is the average total data rate in a sector and the edge throughput is defined as the data rate that 5% of UEs cannot reach [12]. Generally, 5% throughput loss in the victim system is set as the evaluation criterion for the maximum allowable ACI.

### 6.1. Uplink Scheduling in Synchronized Operations

In the UL simulation, MCL of 70 dB including antenna gains is additionally assumed for synchronized operations [12].  Table 8 summarizes reference cell throughput and edge throughput for all TPC sets and scheduling algorithms. The reference values are obtained by assuming no external interference from the aggressor system. The random scheduling allocates one of the three UL channels (16 RBs each) randomly to each UE without considering the ACI distribution. In Table 8, the ACI-based minCL and maxCL scheduling algorithms show higher cell throughput than the random scheduling, whereas the edge throughput is lower. Note that, in the proposed algorithms, a central UE in each sector is assigned the same UL channel as other central UEs in neighboring sectors. Because the central UEs generally transmit with low power, the interference to other central UEs is reduced and thus the cell throughput can be improved. Similarly, in the proposed algorithms, an edge UE in each sector is assigned the same UL channel as other edge UEs in neighboring sectors. The cell throughput of the minCL scheduling is almost the same as that of the maxCL. The edge throughput of the minCL scheduling is slightly higher due to the fast fading characteristics in a low frequency channel. The minCL scheduling allocates the low frequency channel to the edge UE, which can improve the edge UE performance slightly compared with allocation to the high frequency channel. The reference throughput with TPC set 1 is much higher than that with TPC set 2 because UEs with TPC set 1 generally transmit at higher power than those with TPC set 2. TPC set 1 can ensure a higher network throughput in a single system whereas TPC set 2 can reduce the ACI to other systems due to the reduced UE transmit power.

**Figure 7:**
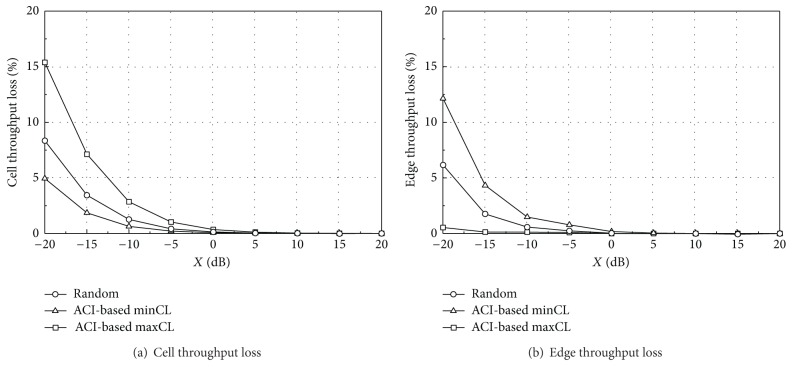
Throughput loss (TPC set 1, coordinated).

**Figure 8:**
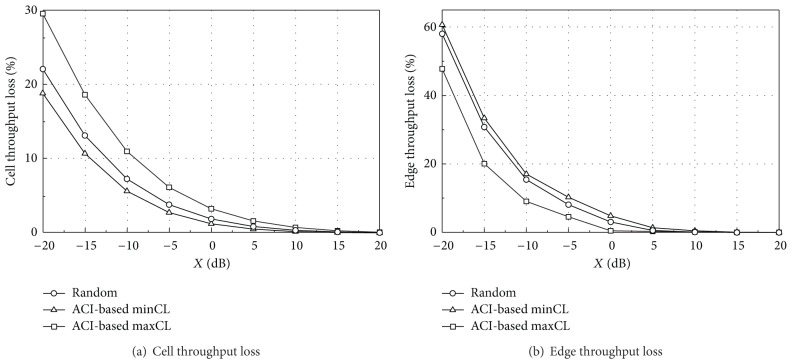
Throughput loss (TPC set 1, uncoordinated).

Figures 7 and 8 give the simulation results for the throughput loss in different network layouts when TPC set 1 is applied to both of the two TDD systems. As the ACI decreases (i.e., a higher value of *X*), the capacity loss decreases to zero. The capacity loss in coordinated layout is much lower than that in uncoordinated layout because the aggressor UEs give much higher interference to the victim BS in uncoordinated layout. Especially, the edge throughput loss is high in uncoordinated layout because more edge UEs in the aggressor system are likely to locate near the victim BS. These aggressor UEs will transmit at a higher power level according to the TPC mechanism, which results in severe interference and edge throughput degradation.

The edge throughput loss of the minCL scheduling is higher than that of other algorithms. The lower frequency band of the victim system is susceptible to interference from the aggressor system and the lower band is allocated to edge UEs in the minCL scheduling. On the other hand, the minCL scheduling has better performance on the cell throughput loss because low-ACI RBs are allocated to the central UEs.

**Figure 9:**
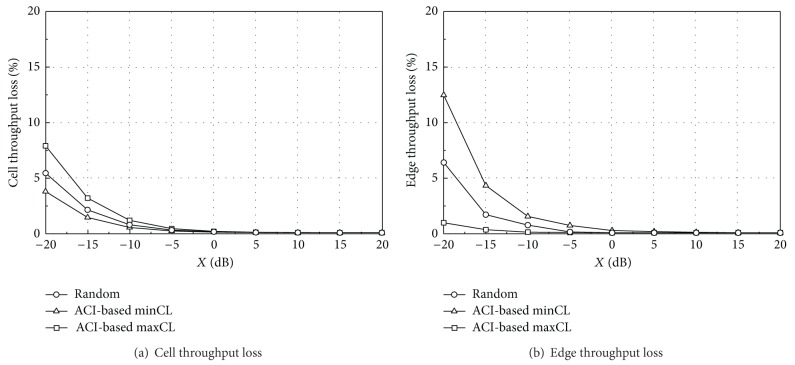
Throughput loss (TPC set 2, coordinated).

**Figure 10:**
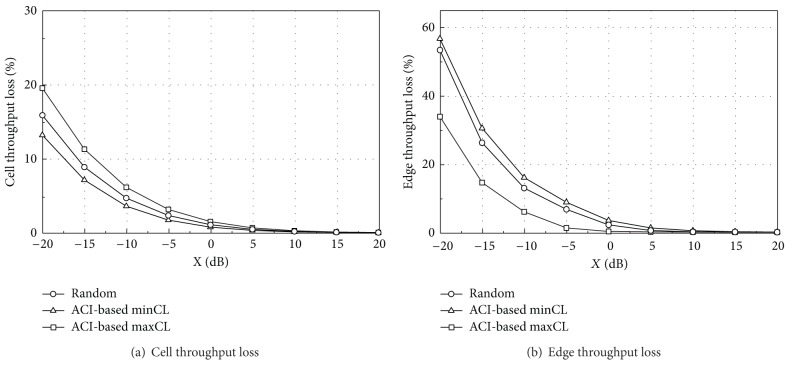
Throughput loss (TPC set 2, uncoordinated).

Figures 9 and 10 show the throughput loss when TPC set 2 is applied. The performance patterns are similar to those of TPC set 1, but the overall throughput loss is much lower due to reduced interference from aggressor UEs. However, as shown in Table 8, TPC set 1 ensures a higher network throughput than set 2.

From Figures 7–10, we can see that each scheduling algorithm is a trade-off between the cell throughput loss and the edge throughput loss. If one algorithm has a higher cell throughput loss, it has a lower edge throughput loss, and vice versa. In coordinated layout, the capacity loss is in an acceptable level for *X* greater than −15 dB and thus there may be no specific preference on the UL scheduling algorithm, from the throughput loss point of view. In uncoordinated layout, the performance is much worse than coordinated layout. The ACI-based minCL and random scheduling algorithms have almost the same edge throughput loss, and the cell throughput loss of the minCL scheduling is lower than that of the random scheduling. The maxCL algorithm shows the highest cell throughput loss among the three algorithms, especially in uncoordinated layout with TPC set 1.

On the other hand, it is worthwhile to compare the absolute throughput performance, not the relative capacity loss. Although the exact values are not shown in this section, they can be easily calculated by combining Figures 7–10 with Table 8. The edge throughput of the random scheduling algorithm is higher than that of other two algorithms for most of *X*. In addition, the minCL algorithm has the highest cell throughput, regardless of the ACIR. From the cell throughput point of view, we recommend the ACI-based minCL algorithm for UL scheduling because the cell throughput is the highest among the three algorithms and is robust against interference from the aggressor system.

### 6.2. ACCL Analysis for Unsynchronized Operations

In this section, the minimum ACCL is obtained in the unsynchronized network and the operational requirements are discussed. The analysis and evaluation procedures for UL and DL are different. The UL interference to a victim BS is caused by a neighboring BS in an adjacent frequency band, whereas the DL interference to a victim UE is caused by a neighboring UE in an adjacent band.

#### 6.2.1. Uplink ACCL Analysis

In the UL unsynchronized operation, the received signal from the nearest BS site can be a main source of interference. According to the proposed ACCL method, it is assumed that only one aggressor BS site with three sectors is located near a victim BS. The UL simulation conditions and parameters are almost the same as the synchronized operation, except the ACI model. The interference to the victim BS is modeled by the received power from the aggressor BS site attenuated by the ACCL. It is assumed that all UL subframes of TD-LTE are exposed on interference from the aggressor BS site in order to take into account the worst-case scenario, where UL reception of the victim BS completely overlaps with DL transmission of the aggressor BS site. Other overlapping scenarios can be extended by utilizing this worst-case result because nonoverlapped subframes have a negligible impact on the throughput loss. 1,000 different UE distribution scenarios are applied to this UL simulation.

**Figure 11:**
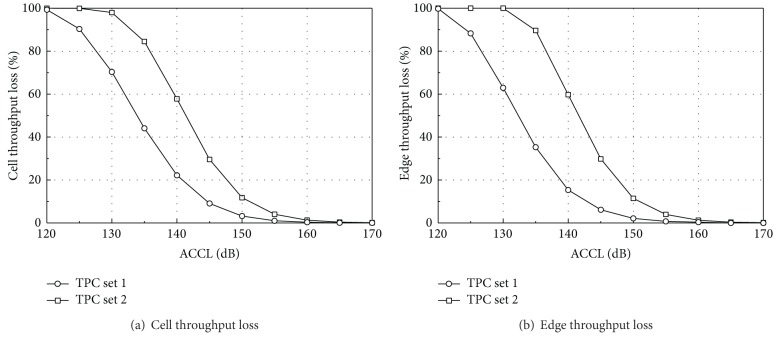
Throughput loss of unsynchronized uplink operations.

Figure 11 shows the UL throughput loss in the unsynchronized operation as a function of ACCL. For 5% capacity loss, the ACCL values of at least 148 dB and 154 dB are required for TPC sets 1 and 2, respectively. By using the minimum ACCL, the related operational requirements for the unsynchronized operation can be provided. First, the ACIR is calculated by the ACLR of the aggressor BS and the ACS of the victim BS. In [17], the recommended ACLR and ACS for BSs are 45 dB and 46.5 dB, respectively, for 10 MHz bandwidth. Thus, from (1), the ACIR is 42.7 dB. Next, the aggressor BS site has a transmit antenna gain of 7.8 to 15 dB considering a 3-sector site structure and antenna directions, whereas the victim BS has a receive antenna gain of 10.2 to 15 dB depending on antenna directions in its serving area. Then, a combined antenna gain has a range of 18 to 30 dB. We set 18 dB as the combined antenna gain assuming appropriate cell-site engineering techniques. Now, considering the ACIR and the combined antenna gain, the minimum PLs between the two BSs are 123.3 and 129.3 dB for TPC sets 1 and 2, respectively. For the BS-to-BS PL, the propagation environment is very different from the ITU-R PL model in (9). In this section, we use the Stanford University Interim (SUI) PL model, which can be applied to BS-to-BS, BS-to-UE, and UE-to-UE cases, with three terrain categories common around the United States [18]. An intermediate PL category is represented by Category B in [18] and a BS antenna height for the transmitter and receiver site is set to 25 m. Then, the median PL for the SUI model can be expressed as follows:

(11) L=78.46+10(4−65ht10000+17.1ht)log10(d100)+6 log10(fc2000)−10.8 log10(hr2),

where *d* is the BS-BS separation in meters (*d* > 100 m), *h*_*t*_ is the transmit antenna height (m), and *h*_*r*_ is the receive antenna height (m). From (11), the PLs of 123.3 and 129.3 dB can be converted to the BS-BS separation of 2,970 m and 4,290 m, respectively.

If only one subframe of TD-LTE overlaps with the DL transmission of the aggressor BS, the overall capacity loss of 5% corresponds to 10% capacity loss in Figure 11. Note that Figure 11 is based on the completely overlapping scenario and TD-LTE is assumed to have two UL subframes. Thus, the minimum ACCL values become 145 and 151 dB for TPC sets 1 and 2, respectively. Assuming the same values of ACIR and the combined antenna gain, the minimum PLs are 120.3 and 126.3 dB, and they correspond to the BS-BS separation of 2,500 m and 3,590 m, respectively. The estimated distance between the two BSs may not be acceptable in an urban network environment, and some special cell-site engineering techniques may be required to reduce the interference. The estimated minimum distance can be different when other PL models and environments are considered.

#### 6.2.2. Downlink ACCL Analysis

In the DL unsynchronized operation, the received signal from the nearest aggressor UE is considered a dominant DL interference source to a victim UE. According to the proposed ACCL method, it is assumed that only one aggressor UE is located near a victim UE and transmits at 23 dBm over entire bandwidth of 10 MHz bandwidth. The DL simulation conditions and parameters are almost the same as the synchronized operation, but the ACI is generated by the aggressor UE near the victim UE. The interference to the victim UE is modeled by the received power from the aggressor UE attenuated by the ACCL. The DL performance is related to a distance between the BS and UE in the victim system, and the same amount of ACI can cause a different impact on the DL performance depending on the distance. Thus, it is reasonable to evaluate the DL performance according to the distance between the BS and the victim UE. For simplicity, each victim UE is located in the direction of the antenna main lobe. In addition, it is assumed that all DL subframes of TD-LTE are exposed to the interference from the aggressor UE. Other overlapping scenarios can be extended by utilizing this worst-case result, as in the UL ACCL analysis.

**Figure 12:**
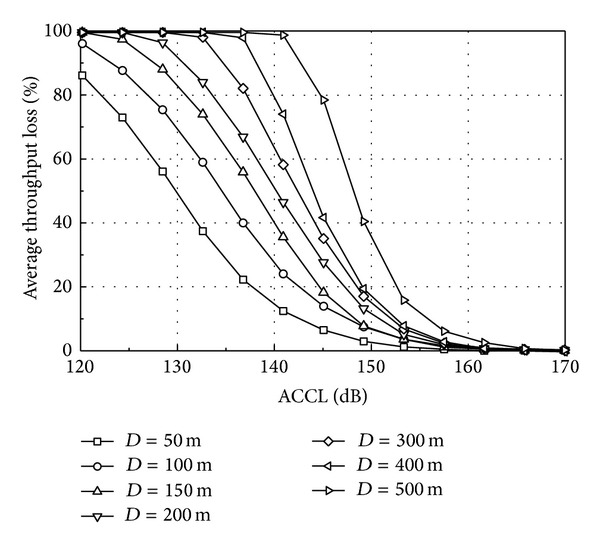
Throughput loss of unsynchronized downlink operations.

Figure 12 shows the DL throughput loss of a victim UE in the unsynchronized operation as a function of ACCL at a different distance (*D*) from the BS. Because one UE is located at a fixed distance from the BS, only the average throughput loss is illustrated in Figure 12 after simulations with 500 different random seeds. The throughput loss decreases as the ACCL increases, and the victim UE far from the BS is very susceptible to the ACI. For *D* = 100, 200, and 500 m, the minimum ACCL values required for 5% throughput loss are 88, 90, and 96 dB, respectively.

By using the minimum ACCL, the related operational requirements can be obtained. First, the ACIR is calculated by the ACLR of the aggressor UE and the ACS of the victim UE. In [19], the recommended ACLR and ACS for UEs are 30 dB and 33 dB, respectively, for 10 MHz bandwidth. Thus the ACIR is 28.2 dB from (1). Because a UE has an omnidirectional antenna, *G*_*a*_ = *G*_*v*_ = 0 dBi. Considering the ACIR and the antenna gain, the minimum PLs for *D* = 100, 200, and 500 m are 59.8, 61.8, and 67.8 dB, respectively. The SUI PL model in (11) may not be accurate if the distance between the transmitter and receiver is less than 100 m. For the UE-to-UE PL, an empirical radio propagation model can be used for low height UE antennas [20]. The model considers line-of-sight (LOS) and nonline-of-sight (NLOS) propagation paths. In this analysis, the LOS model is adopted to consider a high interference scenario and given as follows:

(12) LLOS=4.62+20 log10(4πλ)−2.24ht−4.9hr+29.6 log10(d),

where *d* represents the UE-UE distance (m), *λ* is the wavelength, and *h*_*t*_ and *h*_*r*_ represent the transmitter and receiver height (m), respectively. This empirical model is applicable when both the transmitter and the receiver lie up to 3 m from the local ground. Assuming a UE height of 2 m, the PLs of 59.8, 61.8, and 67.8 dB can be converted to the UE-UE distances of 11, 13, and 21 m, respectively, from (12).

If the UL transmission from the aggressor UE partly overlaps with TD-LTE DL subframes, the required ACCL can be reduced. For example, DwPTS may overlap with WiMax UL as discussed in Section 3. If only DwPTS overlaps with the UL transmission of the aggressor UE, the overall capacity loss of 5% can be inferred from the completely overlapping scenario and it corresponds to 20% loss in Figure 12. Then, the minimum ACCL values are 77, 83, and 89 dB for *D* = 100, 200, and 500 m, respectively, and the minimum PLs are 48.8, 54.8, and 60.8 dB, respectively. They are converted to the distances of 5, 8, and 12 m, respectively.

When compared to the UL unsynchronized operation, the minimum PLs for the DL unsynchronized operation are relatively small. The reason is that the aggressor transmit power (23 dBm) is not as high as the BS (46 dBm), and the omnidirectional antenna is assumed for UEs whereas the BS has directional antennas with high gains. However, mobile operators cannot locate the UE at a specific position to guarantee a minimum distance between two UEs. On the other hand, cell-site engineering may be possible for the UL unsynchronized operation by cooperation between mobile operators.

## 7. Conclusions

In this paper, coexistence issues between TD-LTE and other systems in adjacent spectrum were analyzed and operational requirements were provided with the following research objectives. First, frame synchronization between TD-LTE and WiMax was discussed by investigating possible combinations of TD-LTE and WiMax configurations. TD-LTE configuration 1 can be a strong candidate for coexistence with WiMax because some frame configurations do not overlap with WiMax at all. If UpPTS or DwPTS is disabled, more coexistence candidates can be supported in TD-LTE configurations 1 and 2. Second, an uplink scheduling algorithm was proposed to make the UL transmission robust against the adjacent channel interference by utilizing the interference leakage pattern of transmitters. From the cell throughput point of view, the proposed minCL scheduling is recommendable for UL scheduling because the corresponding cell throughput is higher than that of other algorithms and is robust against interference from the aggressor system. Third, an adjacent-channel coupling loss (ACCL) method was introduced to estimate the minimum requirements for coexistence when two networks are not synchronized. The minimum ACCL can be used to optimize the network parameters and converted to the minimum path loss or the minimum distance between two BSs or two UEs. From the analysis and simulation results, we can see that coexistence of TD-LTE with other systems is feasible if the two networks are synchronized. For the unsynchronized case, the BS-to-BS interference may not be acceptable for a normal operation in an urban environment and some special cell-site engineering techniques may be required to reduce the interference. The UE-to-UE interference is not significant compared to the BS-to-BS interference.

## Figures and Tables

**Table 1:** DwPTS/GP/UpPTS length in a special subframe (OFDM symbols, normal CP).

Format	0	1	2	3	4	5	6	7	8
DwPTS	3	9	10	11	12	3	9	10	11
GP	10	4	3	2	1	9	3	2	1
UpPTS	1	1	1	1	1	2	2	2	2

**Table 2:** TD-LTE DL/UL configuration (D: downlink, U: uplink, S: special subframe).

Configuration	Periodicity	0	1	2	3	4	5	6	7	8	9
0	5 ms	D	S	U	U	U	D	S	U	U	U
1		D	S	U	U	D	D	S	U	U	D
2		D	S	U	D	D	D	S	D	D	D

3	10 ms	D	S	U	U	U	D	D	D	D	D
4		D	S	U	U	D	D	D	D	D	D
5		D	S	U	D	D	D	D	D	D	D

6	5 ms	D	S	U	U	U	D	S	U	U	D

**Table 3:** WiMax frame configuration (TDD mode, 10 MHz).

Configuration	DL symbols	UL symbols	DL duration (ms)	TTG (ms)	UL duration (ms)	RTG (ms)
(35, 12)	35	12	3.600000	0.105714	1.234286	0.06
(34, 13)	34	13	3.497143	0.105714	1.337143	0.06
(33, 14)	33	14	3.394286	0.105714	1.440000	0.06
(32, 15)	32	15	3.291424	0.105714	1.542855	0.06
(31, 16)	31	16	3.188571	0.105714	1.645714	0.06
(30, 17)	30	17	3.085714	0.105714	1.748571	0.06
(29, 18)	29	18	2.982857	0.105714	1.851429	0.06
(28, 19)	28	19	2.880000	0.105714	1.954286	0.06
(27, 20)	27	20	2.777143	0.105714	2.057143	0.06
(26, 21)	26	21	2.674286	0.105714	2.160000	0.06

**Table 4:** Number of TD-LTE symbols overlapped with 10 MHz WiMax (TD-LTE configuration 1).

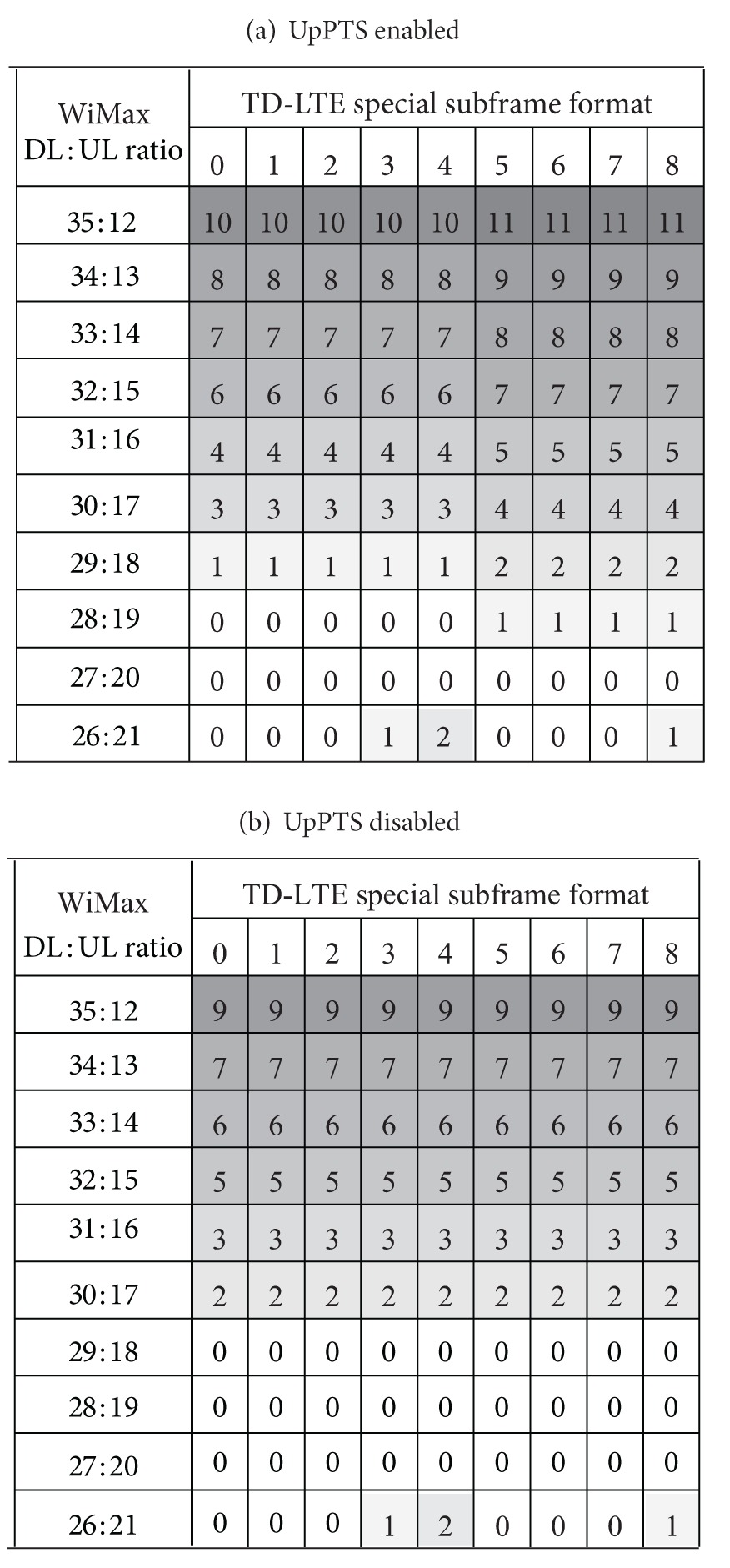

**Table 5:** Number of TD-LTE symbols overlapped with 10 MHz WiMax (TD-LTE configuration 2).

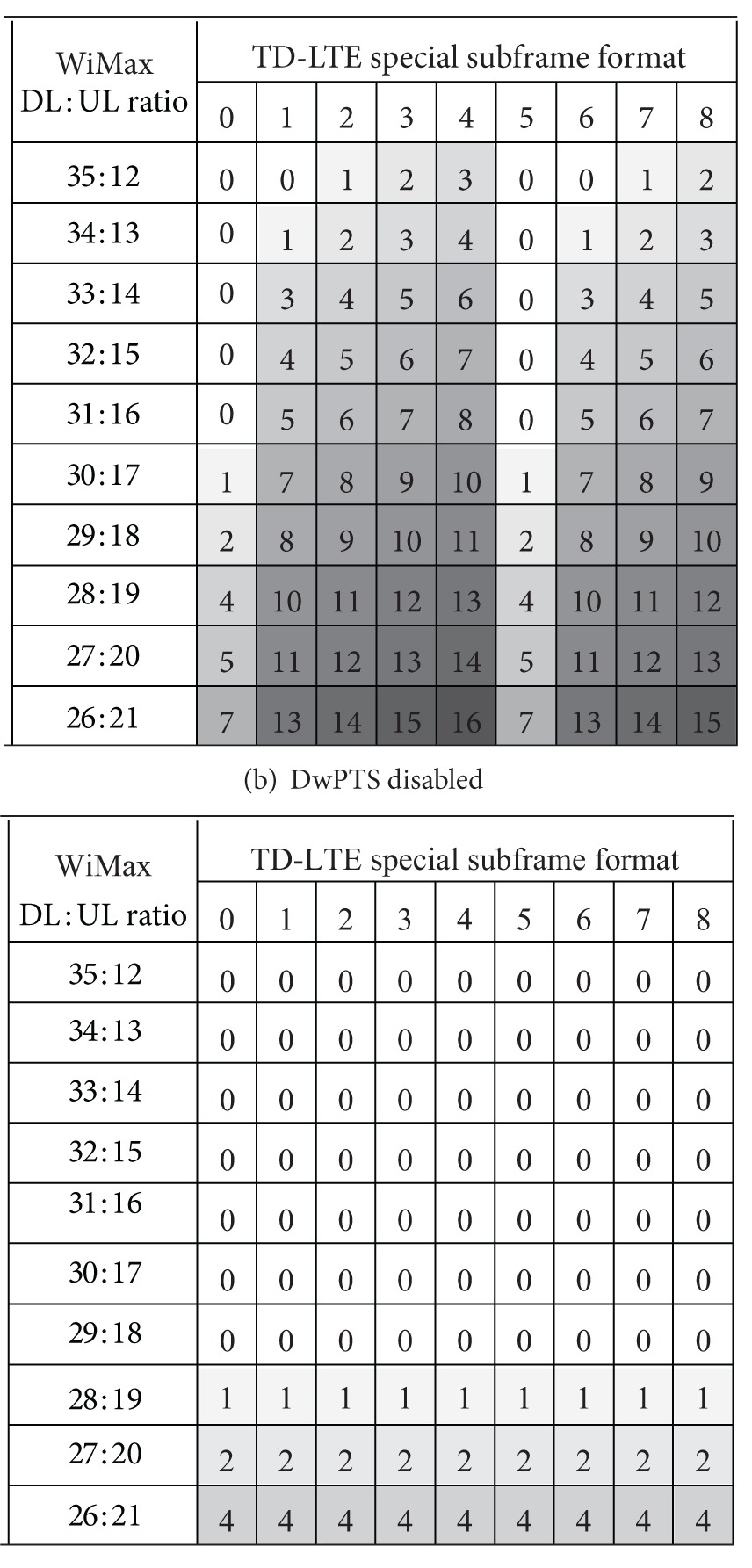

**Table 6:** TPC parameters for TPC sets 1 and 2.

Parameter set	*α*	*P*_0_ (dBm)
Set 1	1	−101
Set 2	0.8	−92.2

**Table 7:** Simulation parameters.

Parameters	Values
Environment	Macro cell, urban area
Carrier frequency	2 GHz
System bandwidth	10 MHz
Noise power density	−174 dBm/Hz
BTS noise figure	5 dB
UE noise figure	9 dB
BTS antenna gain (including feeder loss)	15 dBi
UE antenna gain (omnidirectional antenna)	0 dBi
BS maximum power	46 dBm
UE maximum power	23 dBm
UE minimum power	−40 dBm
Scheduling algorithm	Round robin
Traffic model	Full buffer
TD-LTE configuration	Configuration 1 (DL : UL = 2 : 2)
DwPTS/GP/UpPTS length	10/3/1 symbols (format 2)
Microscale fading	Pedestrian A channel at 3 km/h
DL MIMO receiver modeling	Zero forcing

**Table 8:** Reference cell throughput and edge throughput.

TPC set	Scheduling	Cell throughput	Edge throughput
Set 1	Random scheduling	3.24 Mbps	597 kbps
	ACI-based minCL	3.57 Mbps	538 kbps
	ACI-based maxCL	3.59 Mbps	512 kbps

Set 2	Random scheduling	2.15 Mbps	397 kbps
	ACI-based minCL	2.22 Mbps	361 kbps
	ACI-based maxCL	2.23 Mbps	346 kbps
